# Consumer Attitudes and Use of Antibiotics

**DOI:** 10.3201/eid0909.020591

**Published:** 2003-09

**Authors:** Jodi Vanden Eng, Ruthanne Marcus, James L. Hadler, Beth Imhoff, Duc J. Vugia, Paul R. Cieslak, Elizabeth Zell, Valerie Deneen, Katherine Gibbs McCombs, Shelley M. Zansky, Marguerite A. Hawkins, Richard E. Besser

**Affiliations:** * Connecticut Emerging Infections Program, New Haven, Connecticut, USA; † Connecticut Department of Public Health, Hartford, Connecticut, USA; ‡ Centers for Disease Control and Prevention, Atlanta, Georgia, USA; § California Department of Health Services, Berkeley, California, USA; ¶ Oregon Department of Human Services, Portland, Oregon, USA; # Minnesota Department of Public Health, Minneapolis, Minnesota, USA; ** Georgia Division of Public Health, Atlanta, Georgia, USA; †† New York State Department of Health, Albany, New York, USA; ‡‡ University of Maryland School of Medicine, Baltimore, Maryland, USA

**Keywords:** antibiotic use, antimicrobial resistance, KAP survey

## Abstract

Recent antibiotic use is a risk factor for infection or colonization with resistant bacterial pathogens. Demand for antibiotics can be affected by consumers’ knowledge, attitudes, and practices. In 1998–1999, the Foodborne Diseases Active Surveillance Network (FoodNet) conducted a population-based, random-digit dialing telephone survey, including questions regarding respondents’ knowledge, attitudes, and practices of antibiotic use. Twelve percent had recently taken antibiotics; 27% believed that taking antibiotics when they had a cold made them better more quickly, 32% believed that taking antibiotics when they had a cold prevented more serious illness, and 48% expected a prescription for antibiotics when they were ill enough from a cold to seek medical attention. These misguided beliefs and expectations were associated with a lack of awareness of the dangers of antibiotic use; 58% of patients were not aware of the possible health dangers. National educational efforts are needed to address these issues if patient demand for antibiotics is to be reduced.

Antimicrobial resistance is a rapidly increasing problem in the United States and worldwide. A well-documented risk factor for infection or colonization with resistant bacterial pathogens is recent antibiotic use, particularly within 4 weeks or 1 month before exposure (*1*–*6*). As a result, one of the primary strategies to prevent and control the emergence and spread of resistant organisms is to reduce the selective pressure of overuse and misuse of antibiotics in human medicine (*7*).

Several studies have identified and examined specific causes of the misuse of antibiotics, including unnecessary prescribing (*8*–*14*) and patient demand (*15*–*17*). Factors contributing to inappropriate prescribing practices have been elucidated. In particular, numerous studies of adults have shown that patients’ expectations or physicians’ perceptions of those expectations affect the physicians’ prescribing behavior (*10*,*13*,*16*–*24*).

To solve the problem of antibiotic misuse, a more thorough understanding of what influences the development and expression of patients’ expectations must be gained. Understanding patients’ knowledge, attitude, and practices may facilitate more effective communication between the clinician and patient, as well as aid in the development of strategies to educate patients and the public (*25*). Several lines of evidence suggest educational interventions directed at patients and clinicians can increase patients’ knowledge and awareness, as well as reduce the frequency with which clinicians prescribe antibiotics inappropriately (*26*–*30*).

Our investigation, an analysis of data from a national population-based cross-sectional survey, provides a glimpse of the current knowledge, attitudes, and practices regarding antibiotic use among patients. We also attempt to identify demographic characteristics associated with particular knowledge, attitude, and practices and to determine whether a person’s attitudes toward and knowledge of risks associated with taking antibiotics are associated with recent antibiotic use. Identifying subgroups of the population with high levels of antibiotic use and with misconceptions about antibiotic use will help public health officials target and track the impact of interventions. Other information obtained from this population-based survey will provide with further insight for the development and evaluation of health education and prevention strategies.

## Methods

### Data Source

From February 2, 1998, through February 15, 1999, the Emerging Infections Program’s Foodborne Diseases Active Surveillance Network (FoodNet) conducted a telephone-based population survey in Connecticut, Minnesota, and Oregon, and selected counties in California, Georgia, Maryland, and New York (total population 29 million). Each month, approximately 150 residents in each state were interviewed. After screening to remove business and nonworking telephone numbers, an outside contractor contacted respondents by telephone using a random-digit-dialing, single-stage sampling method (*31*).

These contractors conducted the interviews using methods similar to those used in the Behavioral Risk Factor Surveillance System (*32*). All interviews were conducted in English. Using a standardized questionnaire, they asked one respondent per household about his or her knowledge, attitudes, and recent practices regarding antibiotic use. All members of the household were eligible for selection. Institutional review boards at the Centers for Disease Control and Prevention and all participating states approved the study.

Interviewers obtained verbal consent from all study participants before administering the questionnaire. They informed participants why the information was being collected, and how it would be used, and read them a statement informing them that their participation was voluntary before the start of the interview. No personal identifiers were included in this dataset.

### Survey Questionnaire

Five items (two questions and three statements) addressing participants’ knowledge, attitudes, and practices regarding antibiotic use were included in the survey. Recent antibiotic use referred to antibiotic use in the past 4 weeks. Respondents who took an antibiotic were asked whether the antibiotic was prescribed by their physician for a current illness or for a previous illness or if the antibiotic was prescribed for someone else. For the question, “Are you aware of any health dangers to yourself or other people associated with taking antibiotics?” respondents’ knowledge of health dangers associated with taking antibiotics was classified into the following categories: emerging drug resistance, allergies/reactions, antibiotics may kill “friendly”/“good” microbes, it is unhealthy to take drugs/chemicals in general, misuse/overuse of antibiotics, multiple reasons, other, don't know, or refused. Answers to survey items 1 and 5 were yes/no. For statements 2, 3, and 4, participants were asked to respond according to the following 5-point Likert scale: 1=strongly agree, 2=agree somewhat, 3=unsure, 4=disagree somewhat, and 5=strongly disagree. We classified those who answered “strongly agree” or “agree somewhat” to the antibiotic knowledge questions as having agreed and those who answered “strongly disagree” or “disagree somewhat” as having disagreed. Those who refused to answer a question were not included in the analysis.

In addition to eliciting participants’ responses to these questions, the survey also recorded demographic characteristics of the participants, including their sex, age, income level, education, race, state, and place of residence. Respondents’ place of residence was categorized as urban if they reported living in a city or town of >50,000 residents. Presence of children in the household (yes/no), month of interview, and medical insurance status were also recorded. Respondents were classified as being “with insurance” if they reported any of the following as their type of insurance: health maintenance organization, preferred provider organization, traditional indemnity insurance, Medicaid, Medicare, or other. If respondents reported their type of insurance as “don’t know” or if they refused to answer the question, they were not included in the analysis.

To simplify our analysis, we coded persons indicating Hispanic ethnicity as Hispanic, even if they also identified themselves by race (e.g., a white-Hispanic male would be coded for race as Hispanic). For our multivariable analysis, we grouped persons identified as Asian, Pacific Islander, American Indian, or Alaskan Native into the category called “other.” We also added those who responded “don’t know” or “unsure” to the attitude questions to the “agree” group to divide respondents into two groups: those who responded correctly (disagree) and those who did not (agree or don’t know). For our multivariable logistic regression, we grouped respondents who answered “don’t know” to the question, “Are you aware of dangers associated with antibiotics?” with those who answered “no.” Persons responding “don’t know” to the question, “In the past 4 weeks, have you taken antibiotics?” were not included in the analysis. We evaluated respondents’ education and income levels as continuous variables.

### Statistical Analysis

To compensate for respondents’ unequal probability of selection and allow population estimates to be made, we weighted the data following procedures from the Behavioral Risk Factor Surveillance System (*33*) and based our weighting on the number of residential phone numbers, the number of people per household, and the 1998 postcensus estimates for the age- and sex-specific population of the FoodNet sites (B. Imhoff, pers. comm.). We did not include race in the poststratification weight since some site-sex-age-race groups contained <10 survey participants.

We analyzed the data using SUDAAN (SUrvey DAta ANalysis, v7.5.2, Research Triangle Institute, Research Triangle Park NC), a specialized statistical procedure for analyzing complex sample survey data, and ran the analysis using SAS (Statistical Analysis Software, v6.12) (SAS Institute, Inc., Cary, NC) This software adjusts for the complexity of the sampling design (unequal weighting and clustering) and uses Taylor series linearization methods to estimate variances. Because the ratio of sample size to population size was small, we approximated the sample design by a “with-replacement” design for purposes of variance estimation in SUDAAN. Any bias resulting from such replacement sampling will be in the conservative direction.

We examined respondents’ attitudes toward, and awareness of, antibiotic use by their age, sex, race, income level, education, state, place of residence, medical insurance status, presence of children in household, and month of the interview. We then tested the relationships between respondents’ demographic characteristics and their responses to the questions and statements about antibiotics using chi-square tests for independence. We used the results of the bivariate analyses to develop two multivariable logistic regression models: 1) a model assessing the effects of respondents’ awareness of antibiotic dangers on their attitudes toward and expectations of antibiotics; and 2) a model assessing the influence of respondents’ attitudes on their recent antibiotic use.

Because of the complexity of the analyses, we used only second-degree product terms to assess interaction effects. Results of the logistic regression models are reported as odds ratios (ORs) with 95% confidence intervals (CIs). The level of significance is p=0.05.

## Results

The sample consisted of 12,755 respondents: 7,254 females and 5,501 males. Of these 12,755, a total of 1,975 were <18 years old or of an unknown age and thus were excluded from the analysis (Table 1). Of the remaining 10,780 respondents, 12% reported taking antibiotics within the 4 weeks before the interview (Table 2). Those who took antibiotics within the prior 4 weeks were more likely to be female (13.9% overall, 65% of all who took antibiotics), have medical insurance (12.6%, p<0.01), and live in rural or farm areas (12.9% and 17.6%, respectively, p=0.02). In addition, antibiotic use varied by age group, with the highest use among persons 25–39 years old (13.2%) and those >60 (13.7%) (Figure). We found no significant differences in antibiotic use among groups defined by race, education level, income, state, month of interview, and having children in the household. Of those who took antibiotics (n=1,253), 91% reported using an antibiotic prescribed for a current infection, while 9% reported using an old prescription or someone else’s. No demographic variable was significantly associated with whether respondents used antibiotics obtained to treat their own current illness.

**Table 1:** Demographic characteristics of participants in FoodNet population survey, 1998–1999

Demographic characteristic	N=12,755	%^a^
Sex		
Male	5,501	49.0
Female	7,254	51.0
Age (y)		
<18	1,817	25.4
18–24	1,005	8.9
25–39	3,239	23.5
40–59	4,105	26.1
60 +	2,431	15.4
Unknown	158	0.8
Race		
White	10,278	75.0
Black	1,152	11.2
Hispanic	675	7.6
Asian	339	3.6
American Indian	99	0.9
Other Race	80	0.9
Unknown	132	0.9
Education		
<High school or less	1,792	19.3
High school graduate	3,169	24.7
Some college	3,528	26.4
College graduate	2,556	18.3
Postgraduate	1,595	10.6
Unknown	115	0.8
Income		
<$15,000	1,536	10.6
>$15,000 but <$30,000	2,097	15.5
>$30,000 but <$60,000	3,444	26.1
>$60,000 but <$100,000	1,969	16.2
>$100,000	947	7.8
Unknown	2,762	23.8
Residence		
City/urban	4,374	34.2
Suburban	4,338	33.3
Town/village	1,807	13.4
Rural (not farm)	1,672	14.4
Farm	493	4.3
Unknown	71	0.5
Insurance		
With medical insurance	10,561	79.6
Without medical insurance	990	8.3
Unknown	1,204	12.2

^a^Percentages are based on weighted population data.

**Table 2:** Responses of 10,780 persons to survey items, FoodNet population survey, 1998–1999^a^

Survey item	Yes/agree	No/disagree	Unsure	% Yes
1. In the past 4 weeks, have you (has he/she) taken any antibiotic medicine?	1,255	9,485	N/A	12.0
2. When I have a cold, I should take antibiotics to prevent getting a more serious illness.	2,544	7,638	538	27.4
3. When I get a cold, antibiotics help me to get better more quickly.	3,053	6,758	896	32.2
4. By the time I am sick enough to talk to or visit a doctor because of a cold, I usually expect a prescription for antibiotics.	4,812	4,954	911	47.6
5. Are you aware of any health dangers to yourself or other people associated with taking antibiotics?	4,860	5,749	164	41.9

^a^Values are numbers of persons who answered the questions or statements. Percentages are based on weighted population data.

**Figure:**
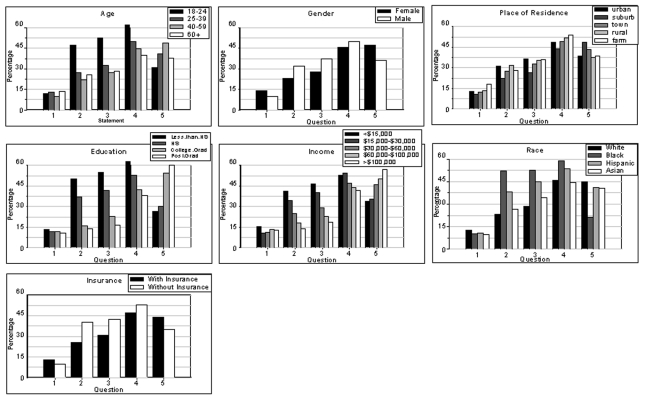
Demographic distributions of responses to five statements about antibiotics. Histograms show the percentage of respondents agreeing with each of the statements. 1) In the past 4 weeks, have you (has he/she) taken any antibiotic medicine? 2) When I have a cold, I should take antibiotics to prevent getting a more serious illness. 3) When I get a cold, antibiotics help me to get better more quickly. 4) By the time I am sick enough to talk to or visit a doctor because of a cold, I usually expect a prescription for antibiotics. 5) Are you aware of any health dangers to yourself or other people associated with taking antibiotics?

Of the 10,780 respondents, 27% believed taking antibiotics when they had a cold prevented more serious illness (survey item 2, Table 2), 32% believed taking antibiotics when they had a cold made them recover more quickly (survey item 3), and 48% expected a prescription for antibiotics when they were ill enough from a cold to seek medical attention (survey item 4). Respondents agreeing with any one of these statements were significantly more likely (p<0.01) to be male, younger (18–24 years), nonwhite, not college educated, and earning <$30,000 per year (Figure). We also found significant differences by place of residence, with respondents living in rural or farm areas being more likely to agree with the statements. Respondents with children were more likely to agree with survey item 2 (28% vs. 26%), item 3 (34% vs. 31%), and item 4 (50% vs. 46%): all differences had p values <0.01. Responses varied among states (p<0.01), with residents of Maryland and Georgia consistently having higher levels of agreement than residents of the other study areas. (For item 2: 27% and 38% vs. 22%–26% [other states] item 3: 35% and 41% vs. 26%–31% [other states], and item 4: 50% and 56% vs. 40%–48% [other states]). Agreeing with the statement, “By the time I am sick enough to see a doctor because of a cold, I usually expect a prescription for antibiotics,” did not vary significantly by month of interview or health insurance status. However, not having insurance was significantly associated with agreement to the statements, “When I get a cold, antibiotics help me to get better more quickly” (42% vs. 27%, p<0.01), and “When I have a cold, I should take antibiotics to prevent getting a more serous illness” (40% vs. 25%, p<0.01). Being interviewed from September through January was also associated with agreeing with these statements (p< 0.05 and p<0.02, respectively).

Fifty-eight percent of respondents were not aware of health dangers associated with taking antibiotics (Table 2). Persons not aware of dangers associated with antibiotic use were significantly (p<0.01) more likely to be male and younger and to live in rural or farm areas. They were also significantly more likely to have less education, lower income, and no insurance (Figure). We found no association between awareness of the dangers of antibiotic use and the month of the interview or having children in the household. Of those aware of health dangers, 58% mentioned factors related to the emergence of drug resistance as a consequence of antibiotic use, 27% mentioned allergies/reactions, 9% recognized that antibiotics kill “good” microbes, and 5% agreed that “it is generally unhealthy to take antibiotics.”

### Multivariable Analysis

#### Associations between Attitude Statements and Awareness of Dangers

We constructed three independent models to assess the relationship between participants’ knowledge of the dangers of antibiotics (and demographic characteristics) and each of the three different attitude statements as the outcome. Each of these relationships was significant in the univariate and multivariable analyses (Table 3).

**Table 3:** Effect of knowledge on attitude statements, FoodNet population survey, 1998–1999^a^

Independent models	Adjusted OR^b,c^	95% CI^d^	
		Upper	Lower
1. Agree that antibiotics prevent serious illness	2.50^d^	2.14	2.92
2. Agree that antibiotics help me get better more quickly	2.29^d^	1.99	2.65
3. Expect a prescription for antibiotics	1.96^d^	1.72	2.23

^a^We constructed three independent models with the three attitude statements as the dependent variables and knowledge of the dangers of antibiotics and selected demographic characteristics as independent variables. ^b^OR, odds ratio; CI, confidence interval. ^c^Adjusted for sex, age, education, race, household income, state, place of residence, and insurance. ^d^Values are significant (p<0.01) after adjusting for multiple comparisons.

Participants not aware of adverse effects of antibiotic use were 2.5 times more likely to agree with the statement, “When I have a cold, I should take antibiotics to prevent getting a more serious illness” (95% CI 2.14 to 2.92). In addition, the demographic variables of age, sex, race, income level, education level, and state were all significant predictors of agreement. We also found significant interactions between the awareness variable and race and education, as well as interactions between age and gender.

We also found a significant association between participants agreeing with the statement, “When I have a cold, antibiotics help me to get better more quickly,” and their being aware of health dangers associated with indiscriminate use of antibiotics (OR 2.29, 95% CI 1.99 to 2.65). Those agreeing with this statement were more likely to be older (40–59 years old: OR 2.20, 95% CI 1.32 to 3.66; and >60 years old: OR 2.08, 95% CI 1.22 to 3.25).

Participants not aware of dangers were 1.96 times more likely to agree with the statement, “By the time I am sick enough to talk to or visit a doctor because of a cold, I usually expect a prescription for antibiotics” (95% CI 1.72 to 2.23). The other demographic variables in the model significantly associated with participants’ responses to this statement were age, sex, income level, education level, insurance, state, and place of residence.

### Association between Antibiotic Use and Attitude Statements and Awareness of Dangers

Using another multivariable model, we examined the association between respondents’ taking antibiotics in the prior 4 weeks and their attitudes toward and knowledge of the adverse effects of antibiotic use (Table 4). The overall model was adjusted for participants’ sex, age, education, race, household income, state, place of residence, child in the house, and insurance. After adjusting for these demographic variables, we found that only one attitude statement remained a predictor of recent antibiotic use. Participants agreeing with the statement, “When I have a cold, antibiotics help me to get better more quickly,” were 1.50 times more likely to have recently taken an antibiotic.

**Table 4:** Effect of attitude and awareness on antibiotic use, FoodNet population survey, 1998–1999^a^

Variable	Adjusted OR^b,c^	95% CI	
		Upper	Lower
Agree that antibiotics prevent serious illness	0.78	0.57	1.06
Agree that antibiotics help me get better more quickly	1.50^d^	1.13	1.99
Expect a prescription for antibiotics	0.96	0.77	1.20
Aware of antibiotic dangers	1.37^d^	1.11	1.69

^a^We constructed a multivariable model to look at the association between respondents taking antibiotics in the previous 4 weeks and their attitudes toward and knowledge about the adverse effects of antibiotic use. ^b^OR, odds ratio; CI, confidence interval. ^c^Adjusted for sex, age, education, race, household income, state, place of residence, child in household, and insurance. ^d^Values are significant (p<0.01) after adjusting for multiple comparisons.

Paradoxically, participants aware of dangers related to antibiotic use were 1.37 times more likely to have taken antibiotics in the previous 4 weeks (95% CI 1.11 to 1.69) even though awareness of these dangers was not a univariate predictor of antibiotic use (OR 0.99, 95% CI 0.49 to 1.98). Of note, only one attitude statement was significant in predicting antibiotic use, suggesting that all of the statements are measuring similar things (Table 4).

## Discussion

The results of this FoodNet survey showed that 12% of adult respondents had used antibiotics during the prior month, most (91%) of which were prescribed for a current infection. Extrapolating from the survey data, we estimate that every adult in the United States in 1998 used antibiotics an average of 1.4 times and that approximately 1 in 10 adults who used antibiotics did so without seeing a physician.

The results also suggest that peoples’ knowledge and attitudes regarding antibiotic use can be substantially improved and that improved knowledge may be important for efforts to reduce the misconceptions and misguided expectations contributing to inappropriate antibiotic use. Overall, 53% of respondents to this population-based survey reported at least one misconception that may put them at unnecessary risk for infection with resistant bacterial pathogens, and 58% were not aware of the health dangers associated with antibiotic use. Nearly half (48%) of the respondents indicated that they expected an antibiotic when they visit a doctor.

This survey identified persons in demographic groups who had both higher levels of misconceptions and lower levels of knowledge about the potential adverse impact of antibiotics. These groups included persons of lower socioeconomic status, lower educational status, males, those in younger age groups, and the elderly. Efforts to reach these groups must be a part of any educational efforts to change patient expectations and to reduce the corresponding pressure on providers to prescribe antibiotics inappropriately.

The results of this study did not show a consistent direct link between misguided expectations and higher levels of recent antibiotic use. In part, this lack may have been due to the design of the survey, which focused on collecting frequency data and did not aim to define the reasons for antibiotic use. In addition, in our analysis, we found that the three attitude statements were similar measures of a person’s opinions on antibiotic use. The statements have the same demographic predictors and association with the knowledge variable and, in reality, they appear to measure the same thing (Table 3).

We did not find an association between recent antibiotic use and lower knowledge levels. Before the analysis, we assumed that persons lacking knowledge about the dangers associated with antibiotic use would be more likely to take antibiotics. However, we found that study participants aware of these health dangers were actually more likely to have taken antibiotics in the prior 4 weeks. Persons of higher socioeconomic status (higher education and income) have better access to health care and are more likely to use antibiotics in general; we did find that people who took an antibiotic recently were more likely to have medical insurance. Another possible explanation is that those who recently took antibiotics may have learned about the adverse effects of antibiotic use from their physician or pharmacist or from their personal experience with antibiotic side effects. Future epidemiologic studies of antibiotic use in diverse populations should be designed to collect information on why participants use antibiotics to distinguish between appropriate and inappropriate antibiotic use.

This type of study has several other important limitations. A telephone survey creates the possibility of selection bias because it may not reflect the population being surveyed (*32*). In addition, the survey catchment population did not include persons who refused to participate, did not have a telephone, did not speak English, or could not respond because of physical or mental impairment. However, the weighting process adjusted for age- and sex-based differences in rates.

Another limitation is the cross-sectional nature of this study. Each participant was assessed only once, and the study was not designed to detect recent changes in opinion. Furthermore, the indicators used measured self-reported behavior not actual behavior. We did not attempt to validate responses on the basis of actual observation, and the survey did not determine whether the antibiotic use was appropriate.

Additionally, respondents may have misunderstood the statements about colds and antibiotics. For example, if they had previous experience with what they thought was a cold, and a physician diagnosed a bacterial ear infection, they may have responded that antibiotics help them get better more quickly when they have a cold (*17*). In addition, several studies have shown that patients often do not have accurate knowledge of antibiotics (*15*,*34*). Hong et al., for example, found that patients often could not identify whether a medication was an antibiotic or not and that many patients considered “antibiotics” to be any prescription medication (*34*).

This study focused only on antibiotic use among adults. Antibiotic use is, however, highest among children, as is the potential for its misuse. In fact, we found that respondents with children in the household were more likely to agree with the attitude statements, demonstrating that it is often parents who influence their children’s perceptions of antibiotic use.

The results of this analysis demonstrate that population-based surveys can contribute to efforts to monitor and reduce inappropriate antibiotic use. The magnitude of recent antibiotic use among adults, as well as widespread lack of awareness about and inappropriate attitudes toward such use indicate that continued population-based surveys could be useful in efforts to monitor trends in antibiotic use. Furthermore, such surveys have the potential to effectively monitor antibiotic knowledge, attitudes, and practices among demographic subgroups of concern. Knowing the magnitude of the problem and the groups who misuse antibiotics most frequently will help public health officials develop and fund intervention efforts, including public information campaigns.

However, our findings also point out some important issues that need to be addressed if this surveillance tool is to be used to full effect. First, additional population-based studies are needed not only to measure antibiotic use but also to determine the reasons that people use them. Such studies should explore the motivations, expectations, and incentives that lead persons to use or not use antibiotics. Second, future studies should include more clearly defined measures of patients’ knowledge. Better measures of knowledge may involve asking respondents to differentiate between antibiotics and other types of prescription medicine and to identify types of infections requiring antibiotics. A more thorough evaluation of respondents’ attitudes may also be useful. To this end, focus groups may help develop questions that better monitor the general population’s attitudes toward antibiotics. Finally, longitudinal tracking of these types of studies will provide important information for the assessment of public health programs.

## References

[1] Goldmann DA, Weinstein RA, Wenzel RP, Tablan OC, Duma RJ, Gaynes RP, Strategies to prevent and control the emergence and spread of antimicrobial-resistant microorganisms in hospitals. A challenge to hospital leadership. JAMA. 1996;275:234–40. 10.1001/jama.275.3.234 8604178

[2] Centers for Disease Control and Prevention. Antibiotic resistance among nasopharyngeal isolates of *Streptococcus pneumoniae* and *Haemophilus influenzae*—Bangui, Central Africa Republic, 1995. MMWR Morb Mortal Wkly Rep. 1997;46:62–4. 9026713

[3] Duchin JS, Breiman RF, Diamond A, Lipman MB, Block SL, Medrick JA, High prevalence of multidrug-resistant *Streptococcus pneumoniae* among children in a rural Kentucky community. Pediatr Infect Dis J. 1995;14:745–50. 10.1097/00006454-199509000-00004 8559622

[4] Nuorti JP, Butler JC, Crutcher JM, Guevara R, Welch D. Holden P, et al. An outbreak of multidrug-resistant pneumococcal pneumonia and bacteremia among unvaccinated nursing home residents. N Engl J Med. 1998;338:1861–8. 10.1056/NEJM199806253382601 9637804

[5] Reichler MR, Rakovsky J, Sobotova A, Slacikova M, Hlavacova B, Hill B, Multiple antimicrobial resistance of pneumococci in children with otitis media, bacteremia, and meningitis in Slovakia. J Infect Dis. 1995;171:1491–6. 7769283 10.1093/infdis/171.6.1491

[6] Magee JT, Pritchard EL, Fitzgerald KA, Dunstan FDJ, Howard AJ. Antibiotic prescribing and antibiotic resistance in community practice: retrospective study, 1996–8. BMJ. 1999;319:1239–40. 10550088 10.1136/bmj.319.7219.1239 PMC28274

[7] Jernigan DB, Cetron MS, Breiman RF. Minimizing the impact of drug-resistant *Streptococcus pneumoniae* (DRSP): a strategy from the DRSP Working Group. JAMA. 1996;275:206–9. 10.1001/jama.275.3.206 8604173

[8] McCaig LF, Hughes JM. Trends in antimicrobial drug prescribing among office-based physicians in the United States. JAMA. 1995;273:214–9. 10.1001/jama.273.3.214 7807660

[9] Kunin CM. Resistance to antimicrobial drugs—a worldwide calamity. Ann Intern Med. 1993;118:557–61. 8442626 10.7326/0003-4819-118-7-199304010-00011

[10] Barden LS, Dowell SF, Schwartz B, Lackey C. Current attitudes regarding use of antimicrobial agents: results from physician’s and parents’ focus group discussions. Clin Pediatr. 1998;37:665–71. 10.1177/000992289803701104 9825210

[11] Gonzales R, Steiner JF, Sande MA. Antibiotic prescribing for adults with colds, upper respiratory tract infections, and bronchitis by ambulatory care physicians. JAMA. 1997;278:901–4. 10.1001/jama.278.11.901 9302241

[12] Nyquist AC, Gonzales R, Steiner JF, Sande MA. Antibiotic prescribing for children with colds, upper respiratory tract infections, and bronchitis. JAMA. 1998;279:875–7. 10.1001/jama.279.11.875 9516004

[13] Schwartz RH, Freij BJ, Ziai M, Sheridan MJ. Antimicrobial prescribing for acute purulent rhinitis in children: a survey of pediatricians and family practitioners. Pediatr Infect Dis J. 1997;16:185–90. 10.1097/00006454-199702000-00004 9041598

[14] Paluck E, Katzenstein D, Frankish CJ, Herbert CP, Milner R, Speert D, Prescribing practices and attitudes toward giving children antibiotics. Can Fam Physician. 2001;47:521–7. 11281085 PMC2018393

[15] Chretien JH, McGarvey M, deStwolinski A, Esswein JG. Abuse of antibiotics. A study of patients attending a university clinic. Arch Intern Med. 1975;135:1063–5. 10.1001/archinte.135.8.1063 1156067

[16] Bauchner H, Pelton SI, Klein JO. Parents, physicians, and antibiotic use. Pediatrics. 1999;103:395–401. 10.1542/peds.103.2.395 9925831

[17] Palmer DA, Bauchner H. Parents’ and physicians’ views on antibiotics. Pediatrics. 1997;99:E6. 10.1542/peds.99.6.e6 9164802

[18] Cockburn J, Pit S. Prescribing behaviour in clinical practice: patients’ expectations and doctors' perceptions of patients’ expectations—a questionnaire study. BMJ. 1997;315:520–3. 9329308 10.1136/bmj.315.7107.520 PMC2127349

[19] Vinson DC, Lutz LJ. The effect of parental expectations on treatment of children with a cough: a report from ASPN. J Fam Pract. 1993;37:23–7. 8345335

[20] Gonzales R, Steiner JF, Lum A, Barrett PH Jr. Decreasing antibiotic use in ambulatory practice: impact of a multidimensional intervention on the treatment of uncomplicated acute bronchitis in adults. JAMA. 1999;281:1512–9. 10.1001/jama.281.16.1512 10227321

[21] Butler CC, Rollnick S, Pill R, Maggs-Rapport F, Stott N. Understanding the culture of prescribing: qualitative study of general practitioners’ and patients’ perceptions of antibiotics for sore throats. BMJ. 1998;317:637–42. 9727992 10.1136/bmj.317.7159.637 PMC28658

[22] Hamm RM, Hicks RJ, Bemben DA. Antibiotics and respiratory infections: are patients more satisfied when expectations are met? J Fam Pract. 1996;43:56–62. 8691181

[23] Macfarlane J, Holmes W, Macfarlane R, Britten N. Influence of patients’ expectations on antibiotic management of acute lower respiratory tract illness in general practice: questionnaire study. BMJ. 1997;315:1211–4. 9393228 10.1136/bmj.315.7117.1211 PMC2127752

[24] Mangione-Smith R, McGlynn EA, Elliott MN, Krogstad P, Brook RH. The relationship between perceived parental expectations and pediatrician antimicrobial prescribing behavior. Pediatrics. 1999;103:711–8. 10.1542/peds.103.4.711 10103291

[25] Kravitz RL, Callahan EJ, Paterniti D, Antonius D, Dunham M, Lewis CE. Prevalence and sources of patients' unmet expectations for care. Ann Intern Med. 1996;125:730–7. 8929006 10.7326/0003-4819-125-9-199611010-00004

[26] Stephenson J. Icelandic researchers are showing the way to bring down rates of antibiotic-resistant bacteria. JAMA. 1996;275:175. 10.1001/jama.275.3.175 8604156

[27] Seppala H, Klaukka T, Vuopio-Varkila J, Muotiala A, Helenius H, Lager K, Finnish Study Group for Antimicrobial Resistance. The effect of changes in the consumption of macrolide antibiotics on erythromycin resistance in group A streptococci in Finland. N Engl J Med. 1997;337:441–6. 10.1056/NEJM199708143370701 9250845

[28] Trepka MJ, Belongia EA, Chyou PH, Davis JP, Schwartz B. The effect of a community intervention trial on parental knowledge and awareness of antibiotic resistance and appropriate antibiotic use in children. Pediatrics. 2001;107:E6. 10.1542/peds.107.1.e6 11134470

[29] Finkelstein JA, Davis RL, Dowell SF, Metlay JP, Soumerai SB, Rifas-Shiman SL, Reducing antibiotic use in children: a randomized trial in 12 practices. Pediatrics. 2001;108:1–7. 10.1542/peds.108.1.1 11433046

[30] Belongia EA, Sullivan BJ, Chyou PH, Madagame E, Reed KD, Schwartz B. A community intervention trial to promote judicious antibiotic use and reduce penicillin-resistant *Streptococcus pneumoniae* carriage in children. Pediatrics. 2001;108:575–83. 10.1542/peds.108.3.575 11533321

[31] Dayton JJ. Proposal to conduct statewide BRFSS for Pennsylvania. Work plan. Burlington (VT): Macro International, Inc.; 1996.

[32] Remington PL, Smith MY, Williamson DF, Anda RF, Gentry EM, Hogelin GC. Design, characteristics, and usefulness of state-based behavioral risk factor surveillance: 1981–87. Public Health Rep. 1988;103:366–75. 2841712 PMC1478092

[33] Gentry EM, Kalsbeek WD, Hogelin GC, Jones Jt, Gaines KL, Forman MR, et al. The behavioral risk factor surveys: II. Design, methods, and estimates from combined state data. Am J Prev Med. 1985;1:9–14. 3870927

[34] Hong JS, Philbrick JT, Schorling JB. Treatment of upper respiratory infections: do patients really want antibiotics? Am J Med. 1999;107:511–5. 10.1016/S0002-9343(99)00270-3 10569308

